# *FADS* Gene Polymorphisms, Fatty Acid Desaturase Activities, and HDL-C in Type 2 Diabetes

**DOI:** 10.3390/ijerph14060572

**Published:** 2017-05-28

**Authors:** Meng-Chuan Huang, Wen-Tsan Chang, Hsin-Yu Chang, Hsin-Fang Chung, Fang-Pei Chen, Ya-Fang Huang, Chih-Cheng Hsu, Shang-Jyh Hwang

**Affiliations:** 1 Department of Public Health and Environmental Medicine and Graduate Institute of Medicine, College of Medicine, Kaohsiung Medical University, Kaohsiung 80708, Taiwan; mechhu@kmu.edu.tw (M.-C.H.); s3223251720@gmail.com (H.-Y.C.); 2 Department of Nutrition and Dietetics, Kaohsiung Medical University Hospital, Kaohsiung 80756, Taiwan; 3 Department of Surgery, School of Medicine, College of Medicine, Kaohsiung Medical University, Kaohsiung 80708, Taiwan; wtchang@kmu.edu.tw; 4 Division of General and Digestive Surgery, Department of Surgery, Kaohsiung Medical University Hospital, Kaohsiung 80756, Taiwan; 5 School of Public Health, University of Queensland, Brisbane, Queensland 4006, Australia; evelyn150@yahoo.com.tw; 6 Department of Nutrition and Health Sciences, College of Health Sciences, Chang-Jung Christian University, Tainan 71101, Taiwan; w787fanny@gmail.com; 7 Institute of Population Health Sciences, National Health Research Institutes, 35 Keyan Road, Zhunan, Miaoli County 35053, Taiwan; avon@nhri.org.tw; 8 Department of Health Services Administration, China Medical University, Taichung 40402, Taiwan; 9 Division of Nephrology, Department of Internal Medicine, Kaohsiung Medical University and University Hospital, Kaohsiung 80708, Taiwan; sjhwang@kmu.edu.tw

**Keywords:** diabetes, *FADS1*, *FADS2*, HDL, genetic polymorphisms, polyunsaturated fatty acids

## Abstract

Polyunsaturated fatty acids (PUFA) correlate with risk of dyslipidemia and cardiovascular diseases. Fatty acid desaturase (*FADS*) single nucleotide polymorphisms (SNPs) modulate circulating PUFA concentrations. This study examined influence of *FADS1* and *FADS2* genetic variants on desaturase activities and blood lipid concentrations in type 2 diabetes patients, and further assessed their interrelationships. Selected SNPs (*FADS1*: rs174547, rs174548, rs174550; *FADS2*: rs174575, rs174576, rs174583, rs498793 and rs2727270) were genotyped in 820 type 2 diabetes patients and compared with those reported in the HapMap. Patient subgroups (*n* = 176) without taking lipid-lowering medicine were studied to assess influence of tag SNPs including rs174547, rs174575, rs498793 and rs2727270 on delta-5 desaturase (D5D: 20:4 (n-6)/20:3 (n-6)) and delta-6 desaturase (D6D:18:3 (n-6)/18:2 (n-6)) activities, and blood lipids. *FADS1* rs174547 TT/TC/CC and *FADS2* rs2727270 CC/CT/TT were significantly (*p* for trend < 0.05) associated with reduced HDL-C, D5D and D6D activities. Upon adjustment for confounders, D5D (*p* = 0.006) correlated significantly and D6D marginally (*p* = 0.07) correlated with increased HDL-C levels, whereas rs174547 and rs2727270 polymorphisms were not associated. D6D andD5D activities may play a role in modulating HDL-C levels in type 2 diabetes. Future studies with larger sample sizes are needed to investigate how *FADS* genetic variations interact with desaturase activities or PUFAs in the metabolism of lipoproteins in diabetic patients.

## 1. Introduction

Type 2 diabetes mellitus is a well-established risk factor for cardiovascular diseases, and is frequently accompanied by metabolic abnormalities including hypertriglyceridemia, high low-density lipoprotein cholesterol (LDL-C) or low high-density lipoprotein cholesterol (HDL-C) [1,2]. Recent studies have implicated a role of raising HDL-C for the prevention and management of diabetes [2,3]. Adults with diabetes have heart disease-related mortality rates that are two to four times higher than those without diabetes [4]. It is, therefore, important to study risk factors for the development of dyslipidemia in patients with type 2 diabetes.

Fatty acid desaturase 1 (*FADS1*) and fatty acid desaturase 2 (*FADS2*) genes are located closely on chromosome 11 (11q12–13.1) [5] and encode delta-5 desaturase (D5D) and delta-6 desaturase (D6D) to [6] serve as rate limiting enzymes for catalyzing the formation of double bonds at delta-5 and delta-6 positions in n-6 or n-3 long chain polyunsaturated fatty acids (PUFA), respectively [7]. Recent gene and genome-wide association studies (GWAS) have shown that polymorphisms in the *FADS* gene cluster have an important impact on lipid profiles and glucose homeostasis, [8,9,10] and thus they play a role in modifying risk of metabolic diseases.

Two recent genome wide association studies have also associated several common variants of *FADS1* and *FADS2* genes with polygenic dyslipidmia [11,12]. Several studies suggested that a cluster of SNPs including rs174547, rs174576 and rs174537are in strong linkage disequilibrium (LD) within the *FADS1-FADS2* region in Caucasian and Asian adults [13,14,15]. SNPs of *FADS1* rs174547 or *FADS2* rs174576 have been correlated with LDL-C [10], HDL-C [11,14] and triglyceride [14]. Furthermore, these polymorphisms also have been shown to modulate D5D and D6D activities, further leading to changes in n-3/n-6 PUFA status [16,17] in healthy adults of different ethnicities.

*FADS* genotypes and desaturase indices have been implicated in insulin resistance [18] and heart diseases [19]. To date, the influences of common *FADS* genetic variants on desaturase activities and blood lipids profile have only been studied in healthy adults of Caucasian or Asian origin, and little research has been undertaken in individuals with diabetes. In this study, we genotyped SNPs (*FADS1*: rs174547, rs174548, rs174550; *FADS2*: rs174575, rs174576, rs174583, rs498793 and rs2727270) in 820 patients with type 2 diabetes and compared our findings with those reported in HapMap in a healthy population. We further assessed influence of tag SNPs including rs174547, rs174575, rs498793 and rs2727270 on D5D and D6D activities and blood lipids in a subgroup (*n* = 176) who were not recorded as receiving lipid lowering drugs on medical records.

## 2. Subjects and Methods

### 2.1. Study Subjects and Inclusion Criteria

We enrolled type 2 diabetes patients who participated in the diabetes management through an integrated delivery system (DMIDS) (ClinicalTrials.gov NCT00288678). Patients between 30 and 70 years old who had been diagnosed or newly diagnosed as having type 2 diabetes by their physicians of primary healthcare clinics based on criteria established by the American Diabetes Association (ADA) were recruited [20]. Pregnant women and participants with a history of myocardial infarction, cerebrovascular accident (e.g., stroke), foot amputation, and uremia under dialysis were excluded at baseline recruitment. The protocol of the study has been described previously [21]. Briefly, 1209 newly diagnosed type 2 diabetes patients were recruited between August 2003 and December 2005. Of those patients, 874 completed the follow-up through the end of 2009. We only included 820 participants who had available DNA collected in year of 2008 for the genotyping that we performed in this study. Furthermore, we selected 176 diabetes patients who, according to their medical records, had not used lipid-lowering drugs (statin and fibrate) and had complete plasma fatty acid workups to examine the associations of concentrations of fatty acids with different polymorphisms of *FADS*. The protocol of this study was approved by the Ethics Committee of the National Health Research Institutes and Kaohsiung Medical University Hospital, Taiwan. Each subject signed an informed written consent form.

### 2.2. Clinical Data Collection and Measurements

Trained research assistants or nurses measured height, weight, as well as systolic and diastolic blood pressure. Venous blood was collected after overnight fasting for at least 8 h for hematological analysis. All blood samples were stored at 2–8 °C and delivered to a central laboratory certified by the College of American Pathology and US Commission on Office Laboratory Accreditation. Glycated hemoglobin (HbA1c) was assessed by high-performance liquid chromatography (Variant II; Bio-Rad Laboratories, Hercules, CA, USA). Levels of plasma blood glucose, triglycerides, cholesterol, LDL-C, HDL-C, and serum creatinine were measured by an automatic analyzer (Hitachi 7060; Hitachi High Technologies, Tokyo, Japan).

### 2.3. Gas Chromatography Analysis of Plasma Lipids

Extraction of plasma lipids was based on a method described by Bligh and Dyer [22]. Extracted lipids were derivatized to fatty acid methyl esters using boron trifluoride-methanol. These were quantified by gas chromatography (6890 N GC system, Agilent Technologies, Santa Clara, CA, USA). The gas chromatographer was equipped with a Agilent J&W DB-225 capillary column (30 m × 0.25 mm inner diameter × 0.25 mm film) with N_2_ being employed as the carrier gas. Fatty acids levels were measured using C17:0 (Sigma, St. Louis, MO, USA) as internal standard and fatty acid concentrations were expressed as a percentage of total area under the peaks (weight %) [23].

### 2.4. Measurements of Desaturase and Pathway Activities

Desaturase and pathway activities were evaluated by ratios of product/precursor fatty acids. Desaturase activities for D6D and D5D were as follows: D6D activity = [18:3 (n-6)/18:2 (n-6)] and D5D activity = [20:4 (n-6)/20:3 (n-6)] [24]. Pathway activities were as follows: n-3 pathway = [20:5 (n-3)/18:3 (n-3)] and n-6 pathway = [20:4 (n-6)/18:2 (n-6)] [15]. 

### 2.5. SNP Selection and Genotyping

*FADS1* SNPs (rs174547, rs174548, rs174550) and *FADS2* SNPs (rs174575, rs174576, rs174583, rs498793 and rs2727270) were chosen based on findings of previous studies [10,11,15]. Four of the SNPs including rs174547, rs174575, rs2727270 and rs498793were tag SNPs with minor allele frequencies (MAF) >5% and were reported in the HapMap of the Han Chinese healthy population. Pairwise |D’| and *r*^2^ values between SNPs were computed using Haploview version 4.2 (Broad Institute, Cambridge, MA, USA). The four SNPs (rs174548, rs174550, rs174576, and rs174583) were genotyped in our study and found to be highly correlated with the tag SNP (rs174547) (*r*^2^ ≥ 0.9). Thus, only the results for the tag SNP (rs174547) are presented.

*FADS* SNP genotyping was analyzed by the GenomeLabTM SNPstream^®^ genotyping platform (Beckman Coulter Inc., Fullerton, CA, USA) and its accompanying SNPstream software suite were used to perform multiplex polymerase chain reaction (12-plex PCR) and SNP genotyping by methods previously described [25]. Primers were designed to amplify DNA, and probes designed to identify the SNPs. Ten percent of the samples were duplicated, masked and tested twice. All SNPs were found to be accurately genotyped at a rate of >95%.

### 2.6. Statistical Analysis

All statistical operations were performed using SPSS version 21.0 (SPSS Inc., Chicago, IL, USA). The normality of the continuous variables was performed using Kolmogorov-Smirnov test. If data were not normally distributed, parameters were transformed logarithmically before analysis. Biochemical data among the *FADS1* and *FADS2* SNP genotypes were compared for significance using independent *t*-test and analysis of variance (ANOVA). Simple linear regression analysis was used to evaluate the trend among *FADS* SNP genotypes (*p* for trend). Multiple linear regression analysis was used to evaluate independent associations among *FADS* polymorphisms, desaturase (D5D and D6D) activities and blood lipids levels by adjusting for confounding factors (age, gender, body mass index, education (≤6 year, >6 year), and diabetes duration (years), HbA1c, alcohol use (yes, no) and exercise (yes, no)). We used the Haploview version 4.2 (Broad Institute, Cambridge, MA, USA) to calculate the pairwise |D’| and *r*^2^ values between SNPs. All statistical results were considered significant at *p* value < 0.05 in a two-sided test.

## 3. Result

### 3.1. General Characteristics of Type 2 Diabetes Patients

The distributions of demographic and clinical characteristics of all type 2 diabetes patients (*n* = 820) and those who did not use lipid-lowering drugs (*n* = 176) are summarized in Table 1. The mean age of the subjects was 59.5 years old and average diabetes duration was 11.0 years. A little more than half (52.6%, *n* = 431) were female.

### 3.2. Genotype and Allele Distribution of FADS1 and FADS2 Polymorphisms among All Type 2 Diabetes Patients

Table 2 shows the distributions of genotype and allele frequencies of the eight selected SNPs in diabetes patients. None of the genotype distributions in our study were differed significantly from those expected under Hardy-Weinberg equilibrium (HWE), except for rs498793. For all SNPs examined in our diabetic subjects, the frequencies of genotypes, rs174547, rs174548, rs174576, rs174583 and rs498793, were comparable to those reported in the HapMap CHD (Chinese in Metropolitan Denver, CO, USA) population.

**Figure 1:**
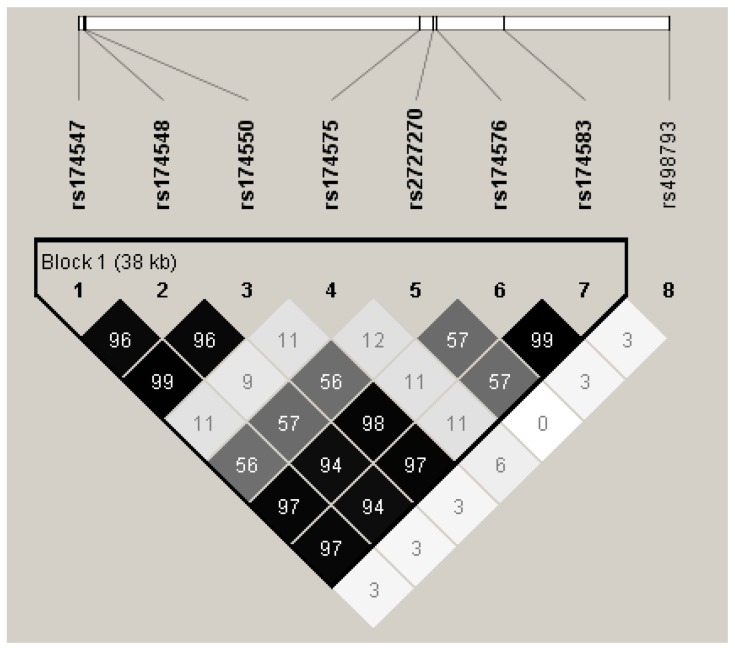
Linkage disequilibrium (LD) block generated by the 8 *FADS*1 and *FADS*2 polymorphisms genotyped, with pairwise *r*^2^ values and color scheme.

As shown in Figure 1, tag SNP rs174547 was in high LD with rs174548 (*r*^2^ = 0.96), rs174550 (*r*^2^ = 0.99), rs174576 (*r*^2^ = 0.97) and rs174583 (*r*^2^ = 0.97). Therefore, only four tag SNPs including rs174547, rs174575, rs2727270 and rs498793 were examined for their impact on desaturase activities (Table 3) and lipid status (Table 4).

### 3.3. FADS1 and FADS2 Genotypes and D5D and D6D Activities in Type 2 Diabetes Patients 

Table 3 presents the desaturase activities categorized by *FADS* genotypes of 176 type 2 diabetes patients not using lipid-lowering drugs. The *FADS1* rs174547 TT, TC, CC genotypes were significantly and linearly associated with reduced D5D (*P for trend* = 0.022) and D6D (*p for trend <* 0.001) activities, as well as with n-3 and n-6 pathway activities (both *p for trend* < 0.001). Similarly, *FADS2* rs2727270 CC, CT and TT genotypes were negatively correlated with D5D (*p for trend* = 0.039), D6D (*p* *for trend* < 0.001), n-3 pathway (*p for trend* = 0.001) and n-6 pathway activities (*p for trend* < 0.001). Subjects carrying rs174575 and rs498793 minor allele were quite few, so genotypes with minor allele were combined for analysis. Subjects with rs174575 CG and GG genotypes (*p* = 0.008) as well as with rs498793 GA and AA genotypes (*p* = 0.031) had significantly lower D6D activities.

### 3.4. FADS1 and FADS2 Genotypes and Lipid Traits in Type 2 Diabetes Patients

Table 4 presents the plasma lipid profiles among *FADS2* genotypes in 176 type 2 diabetes patients not using lipid-lowering drugs. The *FADS1* rs174547 TT, TC, CC (*p* for trend = 0.031) genotypes were negatively associated with reduced HDL-C concentrations. Similarly, *FADS2* rs2727270 CC, CT and TT (*p* for trend = 0.025) genotypes were negatively correlated with HDL-C levels. Frequencies of rs498793 A allele were small, so genotypes with minor allele were combined for analysis. Subjects carrying rs498793 GA and AA genotypes (4.5 ± 0.9 mmol/L) were found to have significantly lower (*p* = 0.034) cholesterol levels than those with GG genotype (4.9 ± 0.9 mmol/L). The *FADS1* rs174575 did not appear to correlate with any lipid trait.

### 3.5. Relations among FADS Genotypes, Desaturases Activities and HDL-C Levels

Table 5 shows the independent associations among *FADS1* rs174547, *FADS2* rs2727270, desaturase activities and HDL-C concentrations in 176 diabetes patients not using statins or fibrates by multiple linear regression analysis. After adjustment for age, gender, body mass index and diabetes duration, HbA1c, alcohol use and exercise habit, model 5 showed that D5D correlated significantly (*p* = 0.006) and D6D was marginally correlated (*p* = 0.07) with increased HDL-C. Whereas rs174547 and rs2727270 polymorphisms were not. We found D5D activities to correlate positively with HDL-C levels and D6D correlate marginally. *FADS1 rs174547* and *FADS2* rs2727270 polymorphisms were not found to correlate with those levels.

## 4. Discussion

This study found *FADS1* rs174547 and *FADS2* rs2727270 genotypes were significantly correlated with decreased D5D and D6D activities and decreased HDL-C concentrations in patients with type 2 diabetes. Since rs174576, rs174548, rs174550 and rs174583 were in strong LD with the tag SNP rs174547, similar significant results were found. Two other studies conducted in Caucasians [13] and Asians [15] have reported SNP of rs174547 in *FADS1* and rs174576 in *FADS2* were in high LD (*r*^2^ ≥ 0.8).

Several earlier studies investigating healthy individuals have also shown that HDL-C concentration can be modified by rs174547 C allele in Japanese [14] and Koreans [26], and by rs174547 C allele and rs174550 T allele of *FADS1* in people of Han Chinese decent [16]. The frequency of rs174547 C allele in our investigation was 0.418, 0.458 in the HapMap Chinese population (Table 2), and 0.31–0.51 in Japanese, Mongolians and Caucasians [11,14]. Furthermore, a genome-wide association study recruiting 19,840 European participants [11] found the minor C allele to be associated with reduced HDL-C (*p* = 2.0 × 10^−12^) and increased triglyceride (*p* = 2.0 × 10^−14^). In that study, the authors measured RNA expression in over 39,000 transcripts from 957 human liver tissues. Performing expression quantitative trait locus analyses, they found the major T allele corresponded to the higher transcript levels (*p* value (*FADS1*) = 5.0 × 10^−35^, *p* (*FADS3*) = 1.0 × 10^−8^). Together, these findings show that SNP of rs174547 impacts both HDL-C levels and mRNA abundance of *FADS1*, suggesting that rs174547 SNP is either functional or in LD with a functional SNP affecting expression or activities of the *FADS1*.

Epidemiological studies have previously estimated D5D and D6D activities using PUFA product-to-precursor ratios. Our study found a reverse relationship between carriers of C alleles of *FADS1* rs174547 and ratios of 20:4 (n-6)/20:3 (n-6) and 18:3 (n-6)/18:2 (n-6) as D5D and D6D desaturase activities as well as 20:4 (n-6)/18:2 (n-6) and 20:5 (n-3)/18:3 (n-3) as n-6 and n-3 pathway activities. Our findings were also confirmed by recent findings of a negative association between rs174547 C allele and reduced desaturase activities or long chain PUFA levels in Dutch [27], in Europeans participating in the HELENA study [24], in Caucasians and Asians participating in Toronto Nutrigenomics and Health study [15] and in elderly Japanese [28]. Although none of these studies directly analyzed desaturase activities, their findings suggest that the C allele of rs174547 may be related to insufficient endogenous conversion of PUFA from their precursors via D5D or D6D.

The biological mechanism underlying how *FADS* genetic variations interact with desaturase activities or PUFAs on HDL-C concentrations remains unclear. It is possible that C alleles of *FADS1* rs174547 may impair desaturases leading to low yields of n-6 and n-3 long chain PUFAs such as 20:4n-6 and 20:5n-3. Furthermore, PUFAs are also known as ligands for peroxisome proliferator activating receptorα,and they might modulate HDL-C metabolism by regulating expression of lipoprotein lipase and apolipoprotein A-I [29] A-II [30] and C-III [31]. 

This study found D5D activity was significantly correlated and D6D was marginally with HDL-C levels, but *FADS1* rs174547and *FADS2* rs2727270 polymorphisms were not, after adjusting for confounding factors. Measurements of plasma n-6 or n-3 PUFA concentrations status represent overall status of PUFA derived from both endogenous synthesis of PUFA via desaturation and elongation as well as exogenous dietary consumption. Although there is evidence that genes for these steps can be regulated by dietary PUFA, the degree to which dietary regulation of longer chain PUFA levels in tissues through changes in expression of desaturase and elongase genes is unclear. Thus, a study based on a larger diabetic sample size is needed to further explore possible interaction between dietary intake of PUFA and genetic variations in *FADS* affecting lipoprotein metabolism. Environmental exposure from diet and smoking is known to impinge on cardiovascular metabolic traits [32]. One study explored the relationship between *FADS1*/*FADS2* SNPs and lipoprotein concentrations in two genetically similar Asian ethnic groups-Japanese (*n* = 21,004) and Mongolia (*n* = 1203) with distinctly different lifestyles [14]. That study found a relationship between C alleles of *FADS1* rs174547 and reduced HDL-C and triglyceride levels in Japanese, but not in Mongolians. Substantial higher plasma n-3 PUFA and higher n-3/n-6 PUFA ratio was observed in Japanese [33] due to the fact that Japanese habitually consume large amounts of fish while Mongolians mainly consume livestock products, which may partly explain the inconsistent impact of rs174547 on blood lipids between these two groups [14]. Hellstrand et al. [10] reported an association between high 18:3n-3/18:2n-6 ratio and 0.04 mmol/L and 0.02 mmol/L higher HDL-C in 4635 Swedish individuals with CC genotype (*p* = 0.046) and TC genotype (*p* = 0.02), but not in those with TT genotype (*p* = 0.96). Their study results indicate that intake levels of n-3/n-6 PUFA modify the associations between genetic variation in *FADS* and HDL-C.

In this study, we did not use multiple test correction to adjust significance of *p* value. While it would be ideal if we could use multiple testing correction to ensure robustness of our analysis, it may be limited in the case of this study because the hypotheses tested in this study were tested by analyses of the a priori, not post hoc, and because we had many comparisons to perform, it would render ideal *p* value indication of significance too small to be achieved. Our study is limited in that our sample was small and our results may not be generalized to diabetes populations of other ethnicities. Future studies need to increase sample sizes of diabetes patient groups and include a healthy controls in order to better examine the relative impacts of *FADS* genetic polymorphisms as well as PUFA status on dyslipidemia in diabetes and healthy subjects.

## 5. Conclusions

In conclusion, *FAD*S1 rs174547 and *FAD*S2 rs2727270 genotypes were significantly correlated with decreased HDL-C concentrations, and D5D /D6D activities as estimated as 20:4(n-6)/20:3 (n-6) and 18:3 (n-6)/18:2 (n-6) in a linear pattern in patients with type 2 diabetes. The results of our multiple variable regression analysis also further revealed that higher HDL-C status levels were positively correlated with higher D5D and marginally with D6D activities, but not with the polymorphisms of FADS1 rs174547and *FAD*S2 rs2727270. Future studies with greater sample sizes are needed to confirm this finding and further explore whether desaturase activities or PUFA status could interact with *FADS* genetic variants to affect lipid metabolism or cardiovascular disease development in type 2 diabetes patients. The understanding of the interaction between environmental modifiers and genetic variants may facilitate future development of preventative strategies for public health genomics and counseling or treatment approaches for personalized medicine.

## Figures and Tables

**Table 1:** Baseline characteristics of 820 type 2 diabetes patients and those who without using lipid-lowering drugs (*n* = 176) ^a^.

Variables	All Type 2 Diabetes Patients (*n* = 820)	Type 2 Diabetes Patients without Using Lipid-Lowering Drugs (*n* = 176) ^b^
Clinical Measures		
Age (year)	59.5 ± 8.5	58.3 ± 8.9
Female (%)	431 (52.6)	99 (56.3)
Diabetes duration (year)	11.0 ± 5.7	10.7 ± 5.3
Education ≤6 years (%)	446 (54.4)	90 (51.1)
Smoking (%)	243 (29.6)	47 (26.7)
Drinking (%)	99 (12.1)	21 (11.9)
Exercise (%)	529 (64.5)	108 (61.4)
Body mass index (kg/m^2^)	26.1 ± 3.8	25.4 ± 3.9
Systolic blood pressure (mmHg)	136.4 ± 19.6	135.5 ± 22.6
Diastolic blood pressure (mmHg)	79.4 ± 11.0	79.1 ± 11.3
Triglyceride (mmol/L)	1.8 ± 1.1	1.6 ± 1.1
Cholesterol (mmol/L)	4.9 ± 0.9	4.8 ± 0.9
LDL-C (mmol/L)	3.1 ± 0.8	3.0 ± 0.8
HDL-C (mmol/L)	1.0 ± 0.3	1.0 ± 0.3
non-HDL-C (mmol/L)	3.9 ± 0.8	3.8 ± 0.9
Fasting glucose (mmol/L)	8.3 ±2.7	8.7 ± 3.0
Hemoglobin A1c (%)	8.0 ± 1.5	8.2 ± 1.6
Serum creatinine (μmol/L)	86.6 ± 67.7	80.7 ± 63.2
Blood urea nitrogen (mmol/L)	5.8 ± 2.8	5.3 ± 2.3
Plasma PUFA (%) ^c^		
Total PUFA		40.5 ± 3.5
n-3 PUFA		6.9 ± 2.2
C18:3 n3 (alpha-linolenic acid)		0.9 ± 0.3
C20:5 n3 (eicosapentaenoic acid)		0.9 ± 0.7
n-6 PUFA		33.6 ± 3.6
C18:2 n6 (linoleic acid)		24.1 ± 4.1
C18:3 n6		0.2 ± 0.2
C20:3 n6		1.7 ± 0.5
C20:4 n6 (arachidonic acid)		6.6±1.6

Abbreviations: LDL-C, low density lipoprotein cholesterol; HDL-C, high density lipoprotein cholesterol. PUFA, polyunsaturated fatty acids; ^a^ Data presented was mean ± SD or n (%). ^b^ 176 type 2 diabetes patients without using lowering lipid drugs (statin and fibrate) and with complete plasma fatty acid measures were selected. ^c^ Only selected n-6 and n-3 PUFA were presented.

**Table 2:** Genotype and allele distribution of *FADS* polymorphisms among 820 type 2 diabetes patients ^a^.

Gene	SNPs	Alleles (Major/Minor)	Genotype (MM/Mm/mm)	Allele Frequency (M/m)	Hapmap Genotype Frequency ^b^ (Allele Frequency)	HWE (*p* Value) ^e^
*FADS1*	rs174547	T/C	280/395/145	955/685	33.3/41.7/25.0 ^c^	0.774
			(34.1/48.2/17.7)	(58.2/41.8)	(54.2/45.8) ^c^	
*FADS1*	rs174548	G/C	268/404/147	940/698	31.8/41.2/27.1 **^c^**	0.806
			(32.7/49.3/17.9)	(57.4/42.6)	(52.4/47.6) ^c^	
*FADS1*	rs174550	C/T	278/397/145	953/687	45.5/40.9/13.6 ^d^	0.874
			(33.9/48.4/17.7)	(41.9/58.1)	(65.9/34.1) ^d^	
*FADS2*	rs174575	C/G	613/188/19	1414/226	80.0/17.8/2.2 ^d^	0.314
			(74.8/22.9/2.3)	(86.2/13.8)	(88.9/11.1) ^d^	
*FADS2*	rs174576	A/C	280/397/143	957/683	31.8/43.5/24.7 **^c^**	0.911
			(34.1/48.1/17.4)	(58.3/41.7)	(53.5/46.5) ^c^	
*FADS2*	rs174583	T/C	280/396/142	956/680	32.1/44/0/23.8 ^c^	0.922
			(34.1/48.3/17.3)	(58.4/42.6)	(54.2/45.8) ^c^	
*FADS2*	rs498793	G/A	683/124/13	1490/150	79.8/20.2/0 **^c^**	0.010
			(83.3/15.1/1.6)	(90.9/9.1)	(89.9/10.1) ^c^	
*FADS2*	rs2727270	C/T	243/421/156	907/733	65.1/25.6/9.3 ^d^	0.270
			(29.6/51.3/19.0)	(55.3/44.7)	(77.9/22.1) ^d^	

Abbreviations: SNP, single nucleotide polymorphism; M, major allele; m, minor allele. ^a^ Data is presented as n (%). ^b^ The Hapmap website (http://www.ncbi.nlm.nih.gov.) provides human genome information based on a general healthy population. ^c^ The HapMap reference was provided by Chinese in Metropolitan Denver (CHD), CO, USA. ^d^ SNPs of rs174550, rs174575 and rs2727270 were not available from the Chinese Han population in Denver but from Han Chinese in Beijing (HCB), China. ^e^ HWE, Hardy-Weinberg equilibrium. HWE for genotype frequency was analyzed by Chi-squared test.

**Table 3:** The differences of fatty acid desaturase activities among *FADS1* and *FADS2* genotypes in type 2 diabetes patients (*n* = 176) ^a^.

Genotypes		*n*	D5D Activity	D6D Activity	n-3 Pathway Activity	n-6 Pathway Activity
			(20:4 n6/20:3 n6)	(18:3 n6/18:2 n6)	(20:5 n3/18:3 n3)	(20:4 n6/18:2 n6)
			Mean ± SD			
*FADS1*	TT	23	4.785 ± 1.162	0.020 ± 0.010	1.001 ±0 .297	0.389 ± 0.087
rs174547 ^d^	TC	90	4.188 ± 1.306	0.011 ± 0.006	0.947 ± 0.358	0.300 ± 0.091
	CC	63	4.126 ± 1.845	0.006 ± 0.005	0.842 ± 0.987	0.233 ± 0.075
	*p* value		0.05	<0.001	0.001	<0.001
	*p* for trend		0.022	<0.001	<0.001	<0.001
*FADS2*	CC	132	4.285 ±1.503	0.011 ± 0.008	0.936 ± 0.716	0.292 ± 0.983
rs174575 ^d^	CG+GG	44	4.120 ± 1.552	0.009 ± 0.007	0.858 ± 0.407	0.266 ± 0.095
	*p* value ^b^		0.426	0.008	0.309	0.098
	*p* for trend ^c^		-	-	-	-
*FADS2*	GG	149	4.199 ± 1.393	0.011 ± 0.008	0.935 ± 0.678	0.291 ± 0.094
rs498793 ^d^	GA+AA	27	4.490 ± 2.069	0.008 ± 0.006	0.815 ± 0.479	0.255 ± 0.113
	*p* value ^b^		0.866	0.031	0.112	0.065
	*p* for trend ^c^		-	-	-	-
*FADS2*	CC	41	4.528 ± 1.300	0.015 ± 0.009	1.039 ± 0.392	0.355 ± 0.101
rs2727270 ^d^	CT	99	4.210 ± 1.477	0.011 ± 0.007	0.923 ± 0.803	0 .275 ± 0.090
	TT	36	4.013 ± 1.802	0.005 ± 0.002	0.759 ± 0.321	0.232 ± 0.069
	*p* value		0.120	<0.001	0.006	<0.001
	*p* for trend		0.039	<0.001	0.001	<0.001

Abbreviations: *FADS*, fatty acid desaturase; ^a^ Data presented is mean ± SD. ANOVA analysis was performed to test the differences among the *FADS* SNP genotypes (*p* value). Simple linear regression analysis was performed to test the trend among *FADS* SNP genotypes (*p* for trend). ^b^ Independent *t*-test analysis was performed to test the differences for SNPs rs174575 and rs498793 genotypes (*p* value). ^c^ Simple linear regression analysis (*p* for trend) didn’t perform as the genotype combined into two groups. ^d^ rs174547, rs174575, rs498793 and rs2727270 are tag SNPs.

**Table 4:** Differences of plasma lipid concentrations among *FADS1* and *FADS2* genotypes in type 2 diabetes patients (*n* = 176). ^a^

	Plasma Lipids						
Genotypes			Triglyceride	Cholesterol	LDL-C	HDL-C	Non-HDL-C
			(mmol/L)	(mmol/L)	(mmol/L)	(mmol/L)	(mmol/L)
		*n*	Mean ± SD				
*FADS1*	TT	23	1.4 ± 0.6	5.1 ± 0.9	3.3 ± 0.9	1.1 ± 0.3	4.0 ± 0.9
rs174547 ^d^	TC	90	1.6 ± 1.2	4.8 ± 0.8	3.0 ± 0.7	1.0 ± 0.2	3.8 ± 0.8
	CC	63	1.7 ±1.1	4.7 ± 1.0	2.9 ± 0.9	1.0 ± 0.3	3.7 ± 0.9
	*p* value		0.699	0.201	0.098	0.098	0.485
	*p* for trend		0.426	0.082	0.059	0.031	0.248
*FADS2*	CC	132	1.5 ± 1.0	4.8 ± 0.9	3.0 ± 0.8	1.0 ± 0.3	3.8 ± 0.9
rs174575 ^d^	CG+GG	44	1.7 ± 1.3	4.9 ± 0.9	3.0 ± 0.9	1.0 ± 0.3	3.8 ± 0.9
	*p* value ^b^		0.625	0.645	0.983	0.964	0.627
	*p* for trend ^c^		-	-	-	-	-
*FADS2*	GG	149	1.6 ± 1.2	4.9 ± 0.9	3.0 ± 0.8	1.0 ± 0.3	3.8 ± 0.9
rs498793 ^d^	GA+AA	27	1.4 ± 0.7	4.5 ± 0.9	2.8 ± 0.8	1.0 ± 0.2	3.5 ± 0.8
	*p* value ^b^		0.482	0.034	0.261	0.298	0.096
	*p* for trend ^c^		-	-	-	-	-
*FADS2*	CC	41	1.5 ± 1.3	5.0 ± 0.9	3.1 ± 0.9	1.1 ± 0.3	3.9 ± 0.9
rs2727270 ^d^	CT	99	1.6 ± 0.9	4.8 ± 0.8	3.0 ± 0.7	1.0 ± 0.3	3.8 ± 0.8
	TT	36	1.6 ± 1.3	4.6 ± 1.0	3.0 ± 1.0	1.0 ± 0.3	3.7 ± 1.0
	*p* value		0.472	0.147	0.249	0.083	0.354
	*p* for trend		0.699	0.059	0.112	0.025	0.299

Abbreviations: LDL-C, low-density lipoprotein cholesterol; HDL-C, high density lipoprotein cholesterol. ^a^ Data presented is mean ± SD. ANOVA analysis was performed to test the differences among the *FADS* SNP genotypes (*p* value). Simple linear regression analysis was performed to test the trend among *FADS* SNP genotypes (*p* for trend). ^b^ Independent *t*-test analysis was performed to test the differences for SNPs rs174575 and rs498793 genotypes (*p* value). ^c^ Simple linear regression analysis (*p* for trend) didn’t perform as the genotype combined into two groups. ^d^ rs174547, rs174575,, rs498793 and rs2727270 are tag SNPs.

**Table 5:** Associations among *FADS1* rs174547, *FADS2* rs2727270, desaturases activities and HDL-C levels type 2 diabetes using multiple linear regression analysis (*n* = 176). ^a^

	Model 1		Model 2		Model 3		Model 4		Model 5	
	Beta (SE)	*p*	Beta (SE)	*p*	Beta (SE)	*p*	Beta (SE)	*p*	Beta (SE)	*p*
rs174547	−0.059 (0.031)	0.056	-		-		-		0.006 (0.050)	0.898
rs2727270	-		−0.057 (0.031)	0.064	-		-		−0.018 (0.045)	0.687
D5D activity	-		-		0.034 (0.014)	0.019	-		0.041 (0.015)	0.006
D6D activity	-		-		-		5.303 (2.759)	0.056	6.239 (3.414)	0.070

Abbreviations: D5D, delta-5-desaturase; D6D, delta-6-desaturase; ^a^ All models are adjusted for confounding factors including age, gender, body mass index, education (≤6 y, >6 y), diabetes duration (years), hemoglobinA1c, alcohol use (yes, no) and exercise (yes, no).

## Abbreviations

The following abbreviations are used in the manuscript:

ANOVA	Analysis and analysis of variance
D5D	Delta-5 desaturase
D6D	Delta-6 desaturase
PUFA	Polyunsaturated fatty acids
*FADS*	Fatty acid desaturase (*FADS*)
HDL-C	High density lipoprotein-cholesterol
HWE	Weinberg equilibrium expectation
LDL-C	Low density lipoprotein-cholesterol
LD	Linkage disequilibrium
MAF	Minor allele frequencies
SNP	single nucleotide polymorphisms
